# How do Typically Developing Deaf Children and Deaf Children with Autism Spectrum Disorder Use the Face When Comprehending Emotional Facial Expressions in British Sign Language?

**DOI:** 10.1007/s10803-014-2130-x

**Published:** 2014-05-07

**Authors:** Tanya Denmark, Joanna Atkinson, Ruth Campbell, John Swettenham

**Affiliations:** 1 Division of Psychology and Language Science, Department of Developmental Science, University College London, London, UK; 2 Deafness Cognition and Language Research Centre, University College London, 49 Gordon Square, London, WC1H 0PD UK

**Keywords:** Autism spectrum disorder, Deafness, Sign language, Emotion, Facial expression

## Abstract

Facial expressions in sign language carry a variety of communicative features. While emotion can modulate a spoken utterance through changes in intonation, duration and intensity, in sign language specific facial expressions presented concurrently with a manual sign perform this function. When deaf adult signers cannot see facial features, their ability to judge emotion in a signed utterance is impaired (Reilly et al. in Sign Lang Stud 75:113–118, 1992). We examined the role of the face in the comprehension of emotion in sign language in a group of typically developing (TD) deaf children and in a group of deaf children with autism spectrum disorder (ASD). We replicated Reilly et al.’s (Sign Lang Stud 75:113–118, 1992) adult results in the TD deaf signing children, confirming the importance of the face in understanding emotion in sign language. The ASD group performed more poorly on the emotion recognition task than the TD children. The deaf children with ASD showed a deficit in emotion recognition during sign language processing analogous to the deficit in vocal emotion recognition that has been observed in hearing children with ASD.

## Introduction

In order to recognize emotions in spoken language, hearing individuals use both visual cues (such as facial expressions and body posture) and auditory cues, such as changes in the frequency, intonation, intensity and rate of speech (Most and Michaelis 2012). However, for deaf individuals, emotional information must be conveyed in sign language using only visual cues. These can be found in the movement and positioning of the hands, face, eyes, torso, shoulders etc. (Vinson et al. 2008). Of these, the range of functions served by the hands and face are the most important (Morgan and Woll 2007; Roberts and Hindley 1999).

Studies examining where deaf individuals look during sign language comprehension have demonstrated that the face is attended to more than other visual cues, including the hands (Agrafiotis et al. 2003; Emmorey et al. 2009). Although there may be a number of reasons for this, for example the face provides linguistic and social information as well as cues for lip reading (Letourneau and Mitchell 2011), one may be that important emotional information is conveyed by the face and that signers need to pay particular attention to facial cues in the absence of tone of voice information and other auditory cues (Reilly et al. 1990).

Surprisingly, only one known study to date has investigated the importance of the face for emotion comprehension in the context of sign language. Reilly et al. (1992) presented deaf adults with video-clips of a model signer producing sentences in American Sign Language (ASL). The content of the sentences was neutral (e.g. _NEXT WEEK MY BROTHER IS COMING TO VISIT_), but the model signer had been asked to produce each sentence with five different emotions: ‘neutral’, ‘happy’, ‘sad’, ‘angry’ and ‘surprised’. In half of the sentences the viewer could see the model signer’s hands and face and in the other half the model signer was wearing a mask so that the face was obscured and only the hands were visible. The viewers were asked to categorize each sentence into one of the five different emotional states labeled on a scoresheet. Although participants were able to use some cues other than the face for emotion recognition in the masked condition e.g. body movement, speed of signing etc. (mean correct response = 77 %), when the face could be seen in the unmasked condition, performance improved significantly (mean correct response = 93 %).

Signers use the face for both emotion recognition and linguistic information. The two systems for emotion and linguistic information have different developmental trajectories and the face is used in qualitatively different ways to express emotion compared with linguistic cues. There are currently no studies with deaf children examining the role of the face in conveying emotional information in sign language. Studies with deaf children have, however, demonstrated a key role for the face in conveying linguistic information. For example Mayberry and Squires (2006) have shown that by 6 years of age, native signing deaf children from deaf families are confidently using facial signs to signify negation and adverbials (Morgan and Woll 2002). In fact, in this group, the development of the use of linguistic expressions parallels that of hearing children. We do not know, though, whether the face is important for emotion comprehension early in the development of sign language or whether it becomes relevant only later when adults become more expert. One of the aims of the current study is to examine the role of the face in emotion comprehension in sign language in a group of typically developing (TD) deaf children.

Studies have shown that hearing children with autism spectrum disorder (ASD) have a particular difficulty interpreting emotions from facial expressions (Grelotti et al. 2002; Grossman and Tager-Flusberg 2012; Hobson et al. 1988; Lacroix et al. 2009; Rump et al. 2009), see Gaigg (2012) for review. The impairments with facial emotion recognition in hearing children with ASD also extend to vocal emotion recognition. Philip et al. (2010) asked adults with ASD and TD controls to identify basic emotions from three conditions: faces, body movements and voices. The ASD group had poorer performance in emotion recognition across all conditions compared to controls. Given evidence of impairment with the face and vocal emotion recognition during language processing in hearing children with ASD, one question for our research was to examine whether the equivalent occurs for deaf children with ASD in the form of a reduced use of the face for emotion comprehension during sign language processing. For this reason we were also interested in making a direct comparison between TD deaf children and deaf children with ASD.

Despite a growing awareness of individuals with a dual diagnosis of deafness and ASD, there have been very few published studies involving this group. Only one study has attempted to systematically estimate the prevalence of ASD in individuals who are deaf. Szymanski et al. (2012) reported that 1.9 % of children in special education in the USA had a diagnosis of hearing loss and ASD. We also know very little about face processing skills in deaf children with ASD. A questionnaire for parents revealed difficulties in using facial expressions and matching facial expressions to actions (Szymanski et al. 2012). However, a number of behaviours one might typically observe in hearing children with ASD were not reported, for example avoidance of eye contact. Parents also reported a higher level of social engagement than in hearing children with ASD, suggesting that the condition might manifest itself differently in children who are also deaf.

Smith et al. (2002) conducted a single case study of a hearing adult with ASD and savant abilities, who had learned British Sign Language (BSL). They noted only a minimal use of facial expressions when signing. While this study provides some clues about emotional expression in sign language in ASD, it neither addresses developmental issues nor the *comprehension* of emotion from a signed utterance.

It is possible that the requirement for all sign users to attend to facial actions may help the deaf child with ASD to interpret facial actions better than might be predicted for a hearing child with ASD. The present study, however, does not explore contrasts between deaf and hearing children with ASD. Instead, we focus on contrasts between TD deaf children and deaf children with ASD. The paradigm developed by Reilly et al. (1992) was used. That study, with deaf adults, found that comprehension of emotion in a (content-neutral) signed utterance was impaired when the face was masked. One motivation for the present study was to examine the extent to which TD deaf children (age range 8.5–16.5 years) showed a similar reliance to that demonstrated for adults on facial actions which are used to interpret emotional meaning in sign. If TD deaf children do show a difference between the interpretation of an emotion from an unmasked and a masked signer, then it is possible that the deaf child with ASD may be less susceptible to face masking (by analogy with the reduced sensitivity to facial actions shown for hearing children with ASD). Whether or not masking affects accuracy of emotion categorization, we predicted (on the basis of results with hearing children with ASD) that the interpretation of emotional meaning in a signed utterance may be less accurate in the child with ASD than TD controls.

## Methods

### Participants

Twelve TD deaf individuals were recruited from deaf schools across the UK. Thirteen deaf individuals with ASD were recruited from the National Deaf Child and Adolescent Mental Health Service, where they had received a diagnosis of ASD from a specialist multidisciplinary social and communication disorders clinic. At this service, deaf individuals are assessed using a number of measures including an ASD diagnostic instrument called the Diagnostic Interview for Social and Communication Disorders, DISCO (Wing et al. 2002); the Leiter-R (Roid et al. 1997) and a play assessment. Diagnosis is given according to information gained from these assessment measures and meets the criteria in the Diagnostic and Statistical Manual of Mental Disorders, Fourth Edition (American Psychiatric Association & American Psychiatric Association. Task Force on DSM-IV 1994). This is currently the most comprehensive assessment for deaf individuals with ASD in the UK. We further confirmed the diagnosis of ASD using the Social Responsiveness Scale (SRS) (Constantino and Gruber 2005) which was completed by each child’s teacher. Teachers of the TD children also completed the SRS to confirm that none of this group had a potential diagnosis of ASD.

All participants had bilateral severe-profound sensori-neural hearing loss. Use of amplification was similar across both groups [ASD group: cochlear implant (7), hearing aids (4) and unaided (1); control group: cochlear implant (4), hearing aids (5) and unaided (3)]. In order to meet the inclusion criteria for the study, participants needed to be able to communicate using sign language at least at a phrasal level. One child in each group was a native signer with deaf parents, the remaining participants were all from hearing families.

The groups were matched for chronological age, non-verbal IQ using the Raven’s Standard Progressive Matrices (SPM) (Raven et al. 1998) and BSL receptive and productive skills, using the BSL Receptive Skills Test, BSLRST (Herman et al. 1999) and the BSL Narrative Skills Test, BSLNST (Herman et al. 2004) (see Table 1).

**Table 1:** Descriptive statistics for the deaf TD and deaf ASD participant groups

Group	Statistic	Age (years:months)	Raven SPM	Raw score percentile		SRS
			Raw scores	BSLRST	BSLNST	Grammar
TD	Mean	12.3	28.4	93.9	40.4	4.8
	SD	2.5	9.3	19.6	21.0	3.7
	Range	8:5–16:5	13–40	56–123	0–75	0–14
ASD	Mean	13:1	28.0	95.7	36.6	68.3
	SD	2:5	10.8	25.2	29.6	34.4
	Range	9:0–17:0	10–46	56–123	10–75	26–141

Independent samples *t* tests indicated no significant difference between the groups in chronological age [*t*(23) = −.716, *p* > .05], Raven’s score [*t*(23) = .103, *p* > .05] on the BSLRST [*t*(21) = .121, *p* > .05] and the grammar percentile on the BSLNST [*t*(19) = .36, *p* > .05].

### Materials

In the present study we used a design based on Reilly et al. (1992) to measure comprehension of facial emotion from BSL sentences in two conditions: when the viewer could see the signer’s (1) face and hands (unmasked face condition), and (2) hands alone (masked face condition) using digital masking. Eight sentences were selected from the 12 sentences in Reilly’s et al.’s experiment (1992)^1^ (see Table 2). We then filmed an experienced deaf BSL signer producing each sentence with a number of different emotions. In addition to the expressions used by Reilly et al. (1992) (‘surprise’, ‘happy’, ‘sad’, ‘angry’ and ‘neutral’), our sign model was also instructed to show ‘annoyance’, ‘disgust’ and ‘mischief’; expressions which are commonly seen and used during language communication. This material formed part of a larger scale study which also explored expressive imitation in deaf children (paper in preparation). We used a single signer for consistency of expressiveness.

**Table 2:** Example sentences

Example sentences BSL–English, from which three sentences were selected per emotion^a^
1. NEXT WEEK POINT SELF BROTHER COME VISIT: Next week my brother is coming to visit
2. DOCTOR NO GIVE MEDICINE: The doctor didn’t give me any medicine
3. FRIEND POINT FOUND DOG WANDER: My friend found her dog wandering
4. POINT SELF MUM GO SHOPPING: My mother has gone shopping
5. GIRL POINT LOOK CAT: The girl is looking for her cat
6. ME ALWAYS EAT MCDONALDS: I always eat McDonalds
7. MUM GIVE NO MONEY: My mum didn’t give me any money
8. WE EAT SALAD LUNCH: We ate salad for lunch

^a^These sentences were signed in BSL, the English translation is provided because English and BSL are different in grammatical structure

**Fig. 1:**
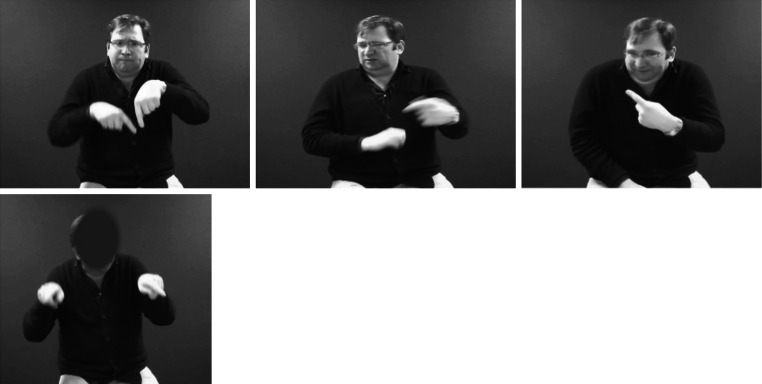
Stills taken from filmed BSL sentences with (from *left* to *right*) angry, disgust and mischief affective facial expressions (and *below*) happy expression with face masked

As in Reilly et al.’s experiment, the sentences were designed to be neutral in content, so that the emotion associated with each sentence could easily be changed by the signer. For example the sentence _‘NEXT WEEK MY BROTHER IS COMING TO VISIT’_ could be produced with a number of emotions such as ‘happy’, ‘sad’ etc. It was therefore not possible for a participant to identify the emotion purely on the basis of the content of the sentence, participants would have to use emotion cues from the BSL clip itself (e.g. facial expression, speed of signing etc.) to discern the emotion conveyed. We paired each of the eight emotions with three BSL sentences totalling 24 emotion sentences (so the same sentence was paired with more than one emotion); these were then presented either with the face displayed (unmasked) (24 items) or with the face digitally masked (another set of 24 items) (see Fig. 1 for an example). In total there were 48 test items, half masked, half unmasked, with six items per emotion (three masked and the same three items unmasked).

### Procedure

Each child was tested individually in a private room at their school. The experimenter (who is a fluent native signer) signed each of the emotion labels (items) on the scoresheet to the child and checked that the participant could give a definition of each. All participants were able to understand the signed sentences in the video and gave adequate descriptions using BSL. Each participant was then given eight practice sentences, where they were shown an example of a BSL sentence performed with each specific emotion and the experimenter explained afterwards “in this sentence the signer was (e.g. happy)” and then pointed to the appropriate written label for the emotion on the scoresheet. The written labels were presented on a sheet of A4 paper ().

Each trial showed one of the 48 video clips of a BSL signed sentence and lasted approximately 8 s. After each trial the participant was asked to indicate which of the eight emotions matched the BSL sentence by either producing the sign for the emotion or pointing to one of the eight written labels on the scoresheet in front of them. The experimenter recorded each response. Test items were presented in a computer generated semi-random order for each participant, with the constraint that the same masking type and the same emotion items could appear no more than twice consecutively.

## Results

One sample *t* tests were calculated for each group to rule out the possibility of participants performing at chance. The *t* tests were calculated on the masked and unmasked condition using a .125 significance level, reflecting eight response choices. Both groups performed significantly above chance on the masked [TD: *t*(11) = 8.2, *p* < .001, ASD: *t*(12) = 5.9, *p* < .001] and the unmasked condition [TD: *t*(11) = 13.3, *p* < .001, ASD: *t*(12) = 7.3, *p* < .001].

**Fig. 2:**
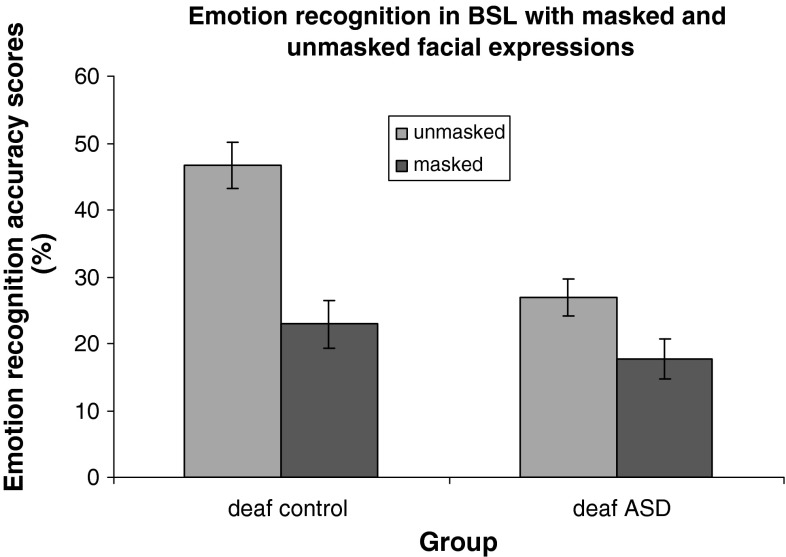
Mean accuracy (%) for masked and unmasked conditions (*error bars* represent SE)

A two-way between-subjects ANOVA was conducted on the accuracy scores, with the factors of diagnostic group (deaf TD vs. deaf ASD) and condition (masked face vs. unmasked face). Mean accuracy for both groups in each condition is shown in Fig. 2.

There was a significant main effect of group F(1, 23) = 11.37, MSE = 1,966.9, *p* < .05 (partial η^2^ = .331). The TD group (M = 68 %) recognised more signed emotions overall compared with the ASD group (M = 46.5 %). There was also a significant main effect of condition F(1, 23) = 38.2, MSE = 3,422.6, *p* < .001 (partial η^2^ = .625), with more emotions recognised in the unmasked condition than the masked condition (deaf TD masked M = 22.9 %, unmasked M = 46.7 %; deaf ASD masked M = 17.6 %, unmasked M = 26.9 %). However, there was also a significant interaction between group and masking condition F(1, 23) = 7.4, MSE = 667.6 *p* < 05 (partial η^2^ = .245), suggesting that the effect of masking the face was greater in the TD group than in the ASD group. Further non parametric tests (Wilcoxon signed-rank; Siegel 1956) demonstrated that there was a significant difference between masked and unmasked conditions both in the deaf TD group z(12) = 2.93, *p* < .003 and the deaf ASD group z(13) = 2.27, *p* < .023. Thus, both groups were sensitive to masking, but the greater magnitude of the difference in the TD group accounts for the significant interaction of group with masking condition.

To further investigate whether the comprehension of specific emotional expressions was impaired or whether deaf individuals with ASD have an overall impairment in emotion recognition relative to deaf controls, both groups were compared on their recognition of specific unmasked emotional expressions. As the distribution across emotion types for both groups was skewed, a Mann–Whitney non-parametric test was used to compare both groups on their recognition of specific affective facial expressions.

A significant difference between groups was found for ‘mischief’ [U(25) = 32.5, *p* < .05, median: deaf TD: 100 %, deaf ASD: 0 %], ‘happy’ [U(25) = 43.0, *p* < .05, median: deaf TD: 100 %, deaf ASD: 33.3 %], and ‘angry’ [U (25) = 30.5, *p* < .05, Median: deaf TD: 66.6 %, deaf ASD: 0 %].

**Fig. 3:**
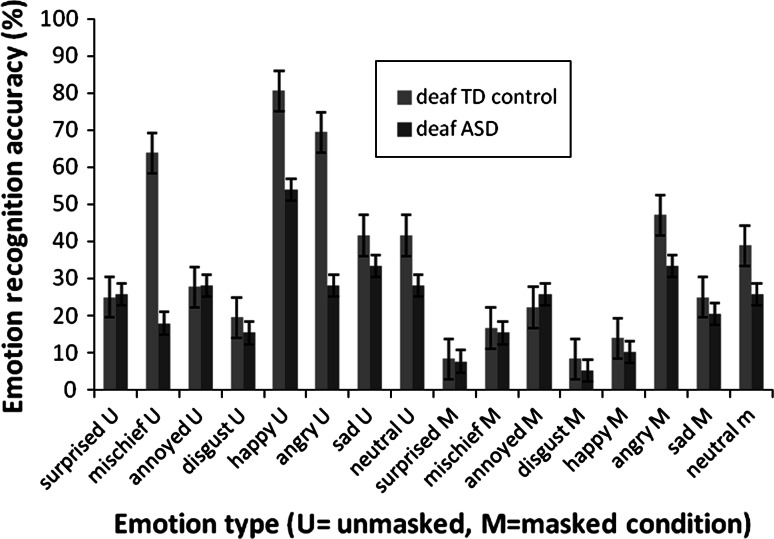
Emotion recognition accuracy scores per emotion type in the masked and unmasked conditions (*error bars* represent SE)

Figure 3 shows that the TD group was significantly better at identifying mischief, happy and angry in the unmasked condition. No significant differences were found for the other five emotions.

Confusion matrices were constructed to examine specific error patterns across respondents, for each target emotion for each condition, for each group. In the unmasked condition, the TD group error pattern (errors significantly different than chance) was as follows: ‘sad’ and ‘neutral’ were confused with each other; ‘surprise’ was confused with ‘happy’, ‘neutral’ and ‘mischief’; ‘annoyed’ was confused with both ‘sad’ and ‘disgust’; ‘disgust’ was confused with ‘sad’ and ‘annoyed’; and ‘mischief’ was confused with ‘happy’. In the ASD group, the pattern was very similar except that the target ‘mischief’ was confused with ‘disgust’, ‘neutral’ and ‘surprise’ as well as ‘happy’.

In the masked condition, ‘happy’ was confused with ‘sad’, ‘neutral’, ‘disgust’ and ‘mischief’ in the TD group, and additionally with ‘angry’ in the ASD group. Similar patterns obtained when ‘sad’, ‘neutral’ and ‘disgust’ were the targets—again, the ASD group additionally used ‘angry’ as a response. That is, when the face was masked, it was difficult to distinguish ‘happy’, ‘sad’, ‘neutral’ and ‘disgust’. The target ‘angry’ was confused with ‘annoyed’ in the TD group, but with both ‘annoyed’ and ‘surprised’ in the ASD group. Finally ‘mischief’ was confused with ‘annoyed’ in the TD group only.

The emotions which fell below chance levels of responding (12.5 %) for both groups were ‘surprise’ and ‘disgust’ but only in the masked condition. For the ASD group ‘happy’ in the masked condition was also below chance levels.

## Discussion

The aim of this investigation was to explore how deaf TD children and deaf children with ASD recognise emotional information in sign language. In the first place, masking of the face affected accuracy of recognition of emotion in sign language. Thus, Reilly et al.’s (1992) finding for adult signers is replicated in children aged 8.5–16.5 years indicating that the use of facial actions to interpret the emotional meaning of a signed utterance is established by this age. The second main finding is that the ASD group were less accurate in their judgments of emotion compared to TD deaf children. The pattern of errors was fairly similar across the two groups, with similar confusions between specific emotions, both for the unmasked and masked conditions, although the ASD group made additional category confusions, especially when ‘mischief’ was the target.

Deaf children with ASD recognised fewer emotions overall on our task when compared with the deaf TD group. That is, the deaf children with ASD showed a deficit in emotion recognition during language processing analogous to the deficit in vocal emotion recognition that has been observed in hearing children with ASD (Philip et al. 2010). Moreover, the significant interaction between group (deaf TD vs. deaf ASD) and condition (masked vs. unmasked) showed that whereas masking the face impaired both groups, the effect was significantly greater for the TD group than the ASD group to the same extent.

The sample was opportunistic due to difficulties recruiting such a rare population, group sizes were small (TD:N = 13, ASD:N = 12) and the age ranges were broad (TD 8.5–16.5 years, ASD 9–17 years). Therefore it was not possible to address more specific questions relating to the precise age when emotional facial expressions start to be accurately recognised, nor whether performance by children and adults is comparable on this particular task. We do not know if the ASD group is delayed or anomalous in their emotion processing from faces.

For typical deaf signers we know that paying attention to the face is important during sign language processing. At the outset of our research we knew very little about whether deaf children with ASD would also use the face in a similar way, as there is an absence of research on deaf individuals with ASD and how they communicate using the face. Our only clue was from hearing ASD children who generally show reduced attention towards faces and impairments in face perception and emotion perception (see e.g. Dawson et al. 2005). Although the ASD group did not benefit from the face to the same extent as controls, they showed a significant effect of masking, suggesting that they make use of some information conveyed by the face during sign language comprehension. It is possible that this may yet prove to be a difference between deaf children with ASD and hearing children with ASD; that is, the requirement for all deaf children to attend to the face in sign language may lead to relatively greater use of the face by deaf than by hearing children with ASD. This prediction could only be tested using very carefully matched groups of deaf and hearing children with ASD.

When comparing the TD and ASD groups on their recognition of individual emotions in the task, both groups responded above chance (12.5 %) for the majority of different emotions, with the exception of ‘surprise’ and ‘disgust’ in the masked condition which were the hardest emotions to identify. ‘Happy’ in the masked condition was also below chance levels of responding but for the ASD group alone. TD children were significantly better at identifying ‘mischief’, ‘happy’ and ‘angry’ in the unmasked condition. For the remaining emotions, performance was similarly low in both groups. The TD group may have shown advantages with identifying ‘happy’ and ‘angry’ emotions, as these tend to be easier to recognise and are acquired earlier in development (Widen and Russell 2003). Previous research demonstrates that hearing individuals with ASD are impaired in understanding ‘surprise’ (Baron-Cohen et al. 1993; Capps et al. 1992; Castelli 2005) and ‘disgust’ (Law Smith et al. 2010). However, in our study, for the displays we used, both the TD and ASD groups responded similarly for these emotions.

An idiosyncratic feature of this study relates to the choice of emotions that were tested. For example, ‘fear’ was not included and neither ‘annoyed’ nor ‘mischief’ feature in Ekman’s criteria for the ‘seven universal basic facial expressions of emotion’ (Ekman 1992). The use of the ‘mischief’ category appears to have been particularly problematic for individuals with ASD. Not only was it significantly less accurate in this group than in TD children, it also generated a wider variety of errors. Thus, in the unmasked condition, while ‘mischief’ was systematically confused with ‘happy’ in TD children, it was confused with several further emotions in the group with ASD. This is in line with their reported difficulties with empathy and attributing mental states to others (Baron-Cohen et al. 2009).

Could the effects reported here reflect factors other than ASD in the deaf children? Since the groups were matched on their BSL receptive and productive skills, and were familiar with the meanings of emotion labels (this was checked before the experiment began) it is unlikely that *linguistic* differences accounted for the findings reported here. An issue for further research is the extent to which specific sign-linguistic features that make use of facial actions, such as eyebrow movements for some intonational aspects, or gaze change to signal role-shift (Dachkovsky and Sandler 2009; de Vos et al. 2009) may be affected by ASD in deaf signers.

Overall accuracy in this study (mean 34.3 %) was considerably lower than for the adults in Reilly et al. (1992) (mean 85 %). While this may reflect a developmental change in performance, it is not possible to directly compare results, as the two studies had different methodologies. We used eight, not five, categories of expression and response choice, which may have made the task considerably harder. We also used a digital mask, imposed after signing had been completed, rather than a physical mask worn by the signing model. One possibility is that the signer in Reilly et al. (1992) may have subtly altered her behaviour when signing while wearing a mask, making recognition of emotions in this condition easier.

### Directions for Future Research

These findings are useful for demonstrating how TD deaf children and deaf children with ASD use emotional information on the face in sign language. A younger sample would highlight developmental trends. The inclusion of children who are as young as 5 or 6 years of age, would tell us more about how children fare with emotional information from the face when they have not yet mastered linguistic uses of facial information in sign language (Morgan and Woll 2002).

Our findings suggest that deaf children with ASD are less accurate in their judgments of emotion compared with TD deaf children. However this does not rule out the possibility that the ASD group use the face effectively for other information (e.g. linguistic information), and this needs to be further investigated. Linguistic facial expressions in sign language differ from emotional facial expressions in important ways; they are more specific in their scope and timing, and are required by the grammar of the language (Corina et al. 1999). Reilly et al. (1990) demonstrated that linguistic facial actions and affective expressions follow different developmental trajectories in the deaf signing child. Predictions for how deaf individuals with ASD would fare with linguistic facial expressions are left open.

Future studies could usefully explore a broader range of measures such as parent/teacher ratings or naturalistic observations in order to get a wider impression of how both deaf individuals with and without ASD comprehend emotional facial expressions in sign language in their everyday lives.

We still do not know whether deaf children with ASD use the face in everyday communication in the same way as TD deaf children. Further studies could measure attention to the face compared with other potential social information.

The issue of whether accuracy of facial expression interpretation is better preserved in deaf ASD than may be expected from a hearing ASD group awaits resolution. Inclusion of a hearing ASD comparison group would be important to highlight whether deafness encourages greater attention to the face in individuals with ASD or whether it leads to a greater impairment of social and communicative skills such as emotion processing.

This is the first attempt to explore how deaf TD children and deaf children with ASD recognise emotions in sign language from the face and other cues. The results provide evidence that TD deaf children who use BSL rely on emotion cues from the face in a similar manner to deaf adults who use ASL. In contrast, deaf ASD children have poorer performance when judging emotional expressions in sign language relative to TD controls, and make more limited use of the face in making emotion decisions. One possible area for intervention with deaf children with ASD would be to teach them explicitly to recognise and be aware of emotional facial expressions in sign language, another would be to train emotion recognition from facial and other visual cues.

## Footnotes

1 Reilly’s experiment was based on ASL. It is important to note that BSL is not mutually intelligible with ASL, since fewer than half of lexical signs are mutually intelligible across the two languages (McKee & Kennedy, 2000). There are some further similarities across these sign languages reflecting the influence of spoken and written English on mouthing and fingerspelling respectively. Therefore some differences may be expected due to the use of ASL in the former and BSL in the current study.
